# Effective pandemic policy design through feedback does not need accurate predictions

**DOI:** 10.1371/journal.pgph.0000955

**Published:** 2023-02-03

**Authors:** Klaske van Heusden, Greg E. Stewart, Sarah P. Otto, Guy A. Dumont

**Affiliations:** 1 School of Engineering, University of British Columbia, Kelowna, BC, Canada; 2 Department of Electrical and Computer Engineering, University of British Columbia, Vancouver, BC, Canada; 3 BC Children’s Hospital Research Institute, Vancouver, BC, Canada; 4 Department of Zoology and Biodiversity Research Centre, University of British Columbia, Vancouver, BC, Canada; Fundacao Oswaldo Cruz, BRAZIL

## Abstract

The COVID-19 pandemic has had an enormous toll on human health and well-being and led to major social and economic disruptions. Public health interventions in response to burgeoning case numbers and hospitalizations have repeatedly bent down the epidemic curve, effectively creating a feedback control system. Worst case scenarios have been avoided in many places through this responsive feedback. We aim to formalize and illustrate how to incorporate principles of feedback control into pandemic projections and decision-making, and ultimately shift the focus from prediction to the design of interventions. Starting with an epidemiological model for COVID-19, we illustrate how feedback control can be incorporated into pandemic management using a simple design that couples recent changes in case numbers or hospital occupancy with explicit policy restrictions. We demonstrate robust ability to control a pandemic using a design that responds to hospital cases, despite simulating large uncertainty in reproduction number *R*_0_ (range: 1.04-5.18) and average time to hospital admission (range: 4-28 days). We show that shorter delays, responding to case counts versus hospital measured infections, reduce both the cumulative case count and the average level of interventions. Finally, we show that feedback is robust to changing compliance to public health directives and to systemic changes associated with variants of concern and with the introduction of a vaccination program. The negative impact of a pandemic on human health and societal disruption can be reduced by coupling models of disease propagation with models of the decision-making process. In contrast to highly varying open-loop projections, incorporating feedback explicitly in the decision-making process is more reflective of the real-world challenge facing public health decision makers. Using feedback principles, effective control strategies can be designed even if the pandemic characteristics are highly uncertain, encouraging earlier and smaller actions that reduce both case counts and the extent of interventions.

## 1 Introduction

With almost 500 million reported cases and over 6.2 million deaths (WHO dashboard, accessed May 17, 2022), the COVID-19 pandemic has placed an enormous burden on individuals, health systems, and society. In order to reduce the rate of transmission of the disease, most countries have implemented similar measures, including lockdowns, closures of restaurants, bars and non-essential services, school closures, curfews, prohibition of large gatherings, obligatory mask-wearing, travel restrictions, testing and tracing, and more recently mass vaccination. All along, public health authorities and governments have had to adjust and modulate those public health measures in response to a very fluid and rapidly evolving dynamic situation. Deciding which intervention to implement and when remains challenging: i) epidemiological models predicting the course of the epidemic show significant uncertainty [1–3] ii) the efficacy of individual measures remains poorly known and may change over time [4], and iii) there is hesitancy to impose strict measures that have significant social, health and economic implications. In order to do so, decision-makers use measures such as daily or weekly incidence rates, hospital admissions, and ICU occupancy rate to set restrictions. They are thus implicitly using feedback. The vast literature on control theory is, however, largely unknown to epidemiologists and public health decision makers.

The aim of this paper is thus to provide the public health community a brief overview of basic feedback control principles and an illustration of how to use them in the decision-making process when designing interventions to manage a pandemic such as COVID-19. Pandemic control is, at its core, not just about disease spread but about behavioural and policy reactions to the disease (i.e., feedback). In the context of the current COVID-19 pandemic, we highlight control principles that could help pandemic management, with the goal of reducing both case counts and socio-economic impacts of restrictions.

**Fig 1:**
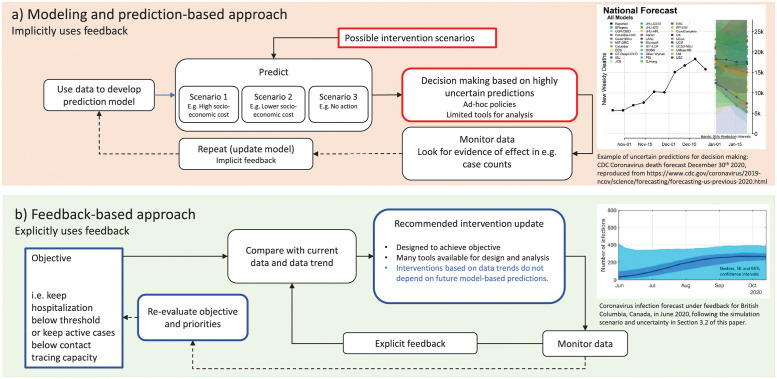
A: Schematic of the widely applied modeling and prediction-based approach to public health policy decision making, illustrating variability in predictions and the implicit feedback used for subsequent decisions. B: Schematic of a feedback-based approach, emphasizing the crucial role of feedback in modifying the spread of the virus.

Explicit consideration of feedback shifts the focus—from prediction to design of interventions. COVID-19 predictions typically show large uncertainty in part because they are scenario based, where one fixed intervention sequence is used to predict possible outcomes (open-loop). In reality, the decision-making process will assess outcomes and adjust accordingly; if the pandemic continues to grow, additional interventions are likely put in place. This decision-making process uses feedback, even if used informally, implicitly, or inconsistently, as illustrated in Fig 1a. In contrast, by representing control of COVID-19 explicitly in a feedback framework as advocated in this paper and illustrated in Fig 1b, the impact of such reactive measures (closed-loop) can be systematically and rigorously analyzed. Instead of implementing ad-hoc policies, transparent and effective policies can be planned and optimized using feedback theory.

Pandemic policy setting involves a process of deciding and communicating interventions to be followed by the public that are intended to deliver a desired reduction in the rate of spread of the virus, often with the goal of managing the case numbers and hospitalizations while protecting the economic and social well-being of the region. These decisions are always made in light of observed case numbers and hospitalizations, which results in a feedback loop, either implicitly as illustrated in Fig 1a or explicitly as illustrated in Fig 1b. This feedback loop plays a critical role in modifying the spread of the virus. A shift to an explicit feedback-based approach acknowledges this critical role of feedback and offers a wide range of tools for policy design and analysis.

The specific suite of interventions considered varies by region and may be approximately ordered from light to severe, given the local context. For example, public indoor mask wearing is typically at the light end of the scale, while full “circuit breaker” lockdowns are at the severe end, with closure of indoor large gatherings somewhere in the middle range. Because objectives and public willingness vary widely among regions, this paper does not aim to suggest a specific set of interventions to control COVID-19. Instead, we aim to emphasize the benefits of using an explicit feedback framework.

While we use a published SEEIQR model [5] to conduct simulations and illustrate these benefits in the face of uncertainty, no such model is needed to implement a feedback policy in practice. We emphasize that effective policy decisions can be made with very limited information about the state and characteristics of the pandemic. Instead of making decisions based on highly uncertain predictions, it is possible to make feedback-guided decisions that will be effective for a very large range of possible pandemic dynamics. Instead of needing accurate information on the state of the pandemic (e.g., about the reproduction number *R*_*t*_) or accurate estimates of the pandemic dynamics, designing effective feedback policies is possible using only approximate models and trend information, even if incomplete.

In addition, we illustrate how feedback mitigates the impact of common concerns such as the impact of delays in measurement and policy implementation, uncertainty on how interventions impact activity levels and spread of the virus, and the fact that only a limited number of interventions are available. We show that such factors limit how well the pandemic can be controlled, but do not limit the applicability of feedback or alter the fundamental properties of feedback control. We show how feedback also reduces the impact of viral evolution, shifts in the efficacy or uptake of a particular intervention, and changes due to vaccination, guiding both the increase and relaxation of interventions.

We show that feedback-guided policies are most effective when delays are minimized and a cumulative feedback measure is taken into account. We show that small early interventions can avoid large interventions later. Hesitancy in implementing control measures risks a greater toll from the disease. Similarly, overly strict measures when the pandemic is under control are unnecessary, adding costs and restricting freedoms. The goal of this paper is to demonstrate how feedback can be harnessed and used to advantage in designing aggressive yet robust public policies even under conditions of high uncertainty. While this will not make difficult decisions easier, it will make them more transparent, informed, and effective.

## 2 Methods

### 2.1 Explicit consideration of feedback

**Fig 2:**
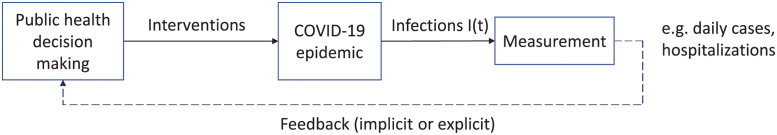
Schematic of public health policy for control of COVID-19 with feedback loop. Public health recommendations that affect COVID-19 infections *I*(*t*) are made using implicit or explicit feedback from measurements such as reported cases or hospitalizations.

The block diagram in Fig 2 shows a schematic of public health policy for control of COVID-19. The pandemic is represented as a black-box, which determines the number of infections *I*(*t*) at any time *t*. This number of infections is unknown, but measurements that are related to this total number of infections are assumed available, such as reported case counts and hospitalizations. As described in the introduction, each jurisdiction will have its own set of interventions available to public policy makers. Here we will abstract those interventions as an input to the pandemic to illustrate the benefits of consistently using feedback principles to affect transmission and manipulate the number of infections. Importantly, we assume throughout that public health measures, if maximized, can prevent spread of a virus. For some highly transmissible variants, this may be considered too costly to achieve, as we have seen in many countries that have decided to let Omicron spread.

By shifting the focus from prediction to design of interventions, feedback gives us a framework for analyzing and designing the public health policy feedback loop. Overly hesitant decision-making -introducing too minor interventions too late- will result in a lack of control over the case numbers. On the other hand, an overly aggressive approach, where interventions swing from light to severe and back again too quickly, is sensitive to model uncertainty and may result in wild oscillations in cases.

There are many techniques developed, deployed, and industrially validated for the design of feedback control systems (see for example Albertos and Mareels [6]). Generally, these techniques allow the designer to balance the speed of control against the harms of overreacting, especially given system noise, delays, and uncertainty about the system to be controlled. While accurate models are more helpful than inaccurate models for the design of feedback controllers, many industrially validated approaches rely on approximate models that can be obtained from simple experiments. Feedback designs that take the resulting uncertainty into account routinely provide reliable control systems in a wide range of safety critical systems.

To illustrate the benefit of an explicit feedback strategy for pandemic control, we select a straightforward control-design technique known as SIMC (simple internal model control), originally developed for process control applications, where dynamics are often complex and nonlinear [7]. We selected SIMC and the particular type of controller it implements (“proportional-integral-derivative” PID) because they are straightforward to describe and to implement, improving the chance of successful application of feedback systems for pandemic control.

The SIMC design method includes the following steps: 1) Obtain a model approximating the process. 2) Depending on the characteristics of the approximate model, the SIMC rules suggest a controller structure and controller parameters as a function of the estimated model parameters. 3) Implement the controller by taking the measured outcome (case counts or hospitalization) as input and using the PID algorithm to compute the interventions that will drive the outcome to its desired value (explicitly using the feedback as indicated schematically in Fig 2).

COVID-19 dynamics are highly uncertain, nonlinear, and measures of the state of the pandemic are delayed and may reflect an unknown proportion of infections. We recommend a logarithmic transformation for feedback, for example the logarithm of daily reported cases or the logarithm of weekly new hospitalizations. This results in a more linear relationship between the interventions and the state of the pandemic. Importantly, this transformation is insensitive to scaling, so it is not necessary to know the exact proportion of infections that are captured in the feedback measure to obtain reliable feedback control and is therefore less sensitive to asymptomatic cases and underreporting.

This simple SIMC control technique will be used to illustrate the impact and characteristics of feedback control under different scenarios using simulations based on an epidemiological model described in Section 2.2. Note that only a simple model approximating the dynamics of the epidemic is required for the design of the controller (e.g., a simple SIR model), which is needed to roughly describe the temporal dynamics at any point in time and is not needed to generate accurate predictions into the future. As a consequence, feedback control can be implemented without a highly accurate epidemiological model because controllers are responsive to deviations from the objective.

### 2.2 Simulation example: An epidemiological model of COVID-19

The advantages of representing control of COVID-19 explicitly in a feedback framework are illustrated using a compartmental SEEIQR (In addition to the standard epidemiological SIR model of susceptibles (S), infected (I), and recovered (R) individuals, the SEEIQR model also includes newly infected individuals without symptoms (non-infectious and infectious, the two E’s) as well as quarantined individuals (Q).) model that was developed to estimate the effect of social distancing in early 2020 in British Columbia, Canada (see Anderson et al. [5] for details and validation). The model was extended to include vaccinations and the appearance of new variants. While we illustrate results based on simulations with one specific COVID-19 model (SEEIQR), similar results were obtained for other COVID-19 models [8], illustrating the value of feedback and the robustness of the proposed feedback control method; a feedback controller designed for one model (the uncertain SEEIQR) also effectively controls cases in structurally different models.

The effect of social restrictions is introduced in this model through the variable *u*(*t*), which we will call “level of activity”, where *u*(*t*) = 0 represents completely restricted social interactions with no transmission and *u*(*t*) = 1 represents normal activity levels. This level of activity *u*(*t*) can vary with time and affects transmission rates. Public health policies are assumed to impact *u*(*t*) through some unknown function that depends on public responsiveness and may change over time.

Next we define the measured variable used to inform the policy decisions. Any measure of infections or hospitalizations will be proportional to the total number of infections *I*_*T*_(*t*) in all groups (i.e., vaccinated, variant, non-variant). The exact number of infected individuals, *I*_*T*_(*t*), is generally not known, but different measures that reflect this state are available, such as daily detected cases and hospitalizations. We assume that these measures, y(t), reflect an unknown fraction of the cases, potentially in a lagged fashion:

$$\begin{array}{c} y\left(t\right)=p\cdot I_{T}\left(t-d\right) \end{array}$$ (1)

where *p* represents the (unknown) fraction of the infected population that is detected and *d* represents the delay in days before measurement. In this paper we will contrast two different measures. In Scenario 1 we consider tracking hospital occupancy, and we set *p* = 0.073 and a delay of *d* = 14 days, as estimated for COVID-19 [9].

$$\begin{array}{c} y_{h}\left(t\right)=0.073\cdot I_{T}\left(t-14\right) \end{array}$$ (2)

Scenario 2 focuses instead on tracking new cases with fast public testing, where we set a much shorter delay of *d* = 2 days, and an unknown fraction *p* of detected cases:

$$\begin{array}{c} y_{t}\left(t\right)=p\cdot I_{T}\left(t-2\right) \end{array}$$ (3)

### 2.3. Systematic feedback design of interventions

#### 2.3.1 Approximate model for design

While we will simulate the performance of feedback control against the full nonlinear SEEIQR model, the design of the control policy uses a simple linear approximation of that complex model. This linear model approximates the pandemic dynamics and response to interventions.

In a first step in the model approximation, we select a linearization point *S*(*t*) = *S*_0_, which is commonly used to linearize epidemiological models and may overestimate transmission when the number of susceptible individuals decreases substantially. The relationship between the social activity level, *u*(*t*), and the measure of cases, *y*(*t*), remains nonlinear.

Second we transform the measured variable to a logarithmic scale *z*(*t*) = ln(*y*(*t*)) and the intervention variable to *v*(*t*) = *u*(*t*) − *u*_0_, where *u*_0_ is the unknown level of restrictions required to achieve zero pandemic growth. This results in an approximate linear relationship in the dynamics relating transformed inputs *v*(*t*) and transformed states *z*(*t*) and is insensitive to scaling. For the special case of a simple one-group SIR model the transformation leads to an exactly linear relationship, see S3 Appendix.

Lastly, a simple approximate model with three parameters is estimated from the response of the complete, nonlinear SEEIQR model (S1 Appendix). The SEEIQR model is injected with a series of steps in intervention *u*(*t*) to obtain an input-output response of the transformed variables *z*(*t*) and *v*(*t*) over a range of operating points and a linear dynamic model (In practice, the fraction p of infections that are detected is often not known. However, as long as this fraction p is (piecewise) constant, it does not affect the linearized model (due to the log transformation above), and the scaling does not need to be known to implement effective control strategies (see S3 Appendix for details).) fit to the response using a non-linear least squares estimation (the output-error method [10]). The resulting model is given by:

$$\begin{array}{c} \frac{d^{2}z\left(t\right)}{dt^{2}}=A\frac{dz(t)}{dt}+B\cdot v\left(t-d\right) \end{array}$$ (4)

with constants *A* = −0.5787 and *B* = 0.1572 for the parameters of model (7)-(9), as described in S3 Appendix. This model is a simple approximation to the nonlinear SEEIQR model yet is sufficient to design a feedback controller for the effective control of the full nonlinear SEEIQR system as illustrated below.

#### 2.3.2 Design of interventions

We will assume that pandemic intervention policies are defined by a function:

$$\begin{array}{c} u(t)=f(y(t)) \end{array}$$ (5)

meaning that there is some rule, *f*(), that translates the measured variable *y*(*t*), reflective of the number of infected cases *I*_*T*_(*t*), into public health measures, *u*(*t*), that affect transmission. The formulation in (5) is general enough to cover most policies, whether ad hoc heuristics or more systematic approaches. Although for illustration purposes we mainly explore cases where there is a nearly continuous set of potential measures, we also show that effective control can be achieved even when a finite set of measures is available.

**Fig 3:**
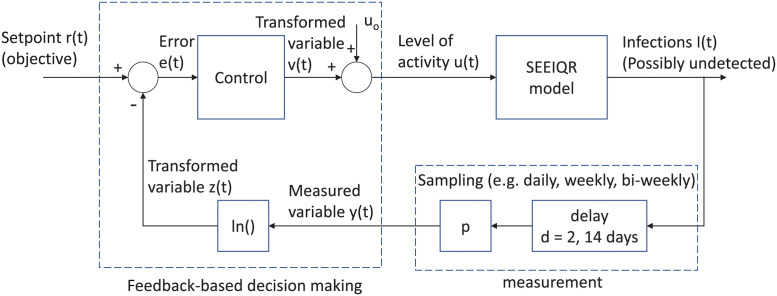
This block diagram illustrates the feedback configuration used in our simulations where the SEEIQR model (see S1 Appendix) is used to simulate the spread of COVID-19. The complete simulated feedback loop includes the SEEIQR model, measurements of case counts (1), the feedback control (6), and all transformations described in Section 2.

The goal is then to design this function such that the resulting closed-loop delivers the desired performance and robustness with respect to unknown pandemic characteristics. An overview of the feedback configuration considered in our simulation example is given in Fig 3.

For systems such as the COVID-19 pandemic where the response delay, *d*, is significant, the SIMC method [7] recommends a proportional-integral control policy:

$$\begin{array}{c} v\left(t\right)=K_{p}\cdot e\left(t\right)+K_{i}\cdot \int_{t_{0}}^{t}e\left(\tau \right)d\tau \end{array}$$ (6)

where *e*(*t*) = *r*(*t*) − *z*(*t*) is the error between the feedback values *z*(*t*) and their desired value, setpoint *r*(*t*) (e.g. set to a number of cases that is smaller than the healthcare capacity of the region and may be varied over time). The format of this controller is the structure used in over 90% of control engineering applications [11]. The integrator in the second term in feedback controller (6) has the desired effect of driving measured outputs to the setpoint *z*(*t*) = *r*(*t*) in spite of model uncertainty. Implicit feedback systems, as used to manage COVID-19 in most jurisdictions, do not use an integrator (*K*_*i*_) but respond directly to measures such as current case counts. As we shall see below, this results in less effective control. The constants *K*_*p*_ and *K*_*i*_ are tuning parameters that can be used to adjust the speed of response, balancing aggressiveness and robustness (S2 Appendix).

## 3 Results

With the closed-loop system defined above and illustrated in Fig 3, we examine real-world scenarios of common concern, using simulations of an SEEIQR model to explore the impact of uncertainty in epidemiological model parameters and the role of time delays in case counts. We illustrate the impact of different policy update frequencies. We address uncertainty on the correspondence between interventions and spread of the virus, including simulating changing public compliance. We also address the fact that only a limited number of interventions may be available by simulating discrete interventions. Finally, we illustrate how feedback-based decision making limits pandemic spread, even as new variants evolve, and can be used to guide reduction of interventions when vaccinations become available.

### 3.1 Analysis of system properties

**Fig 4:**
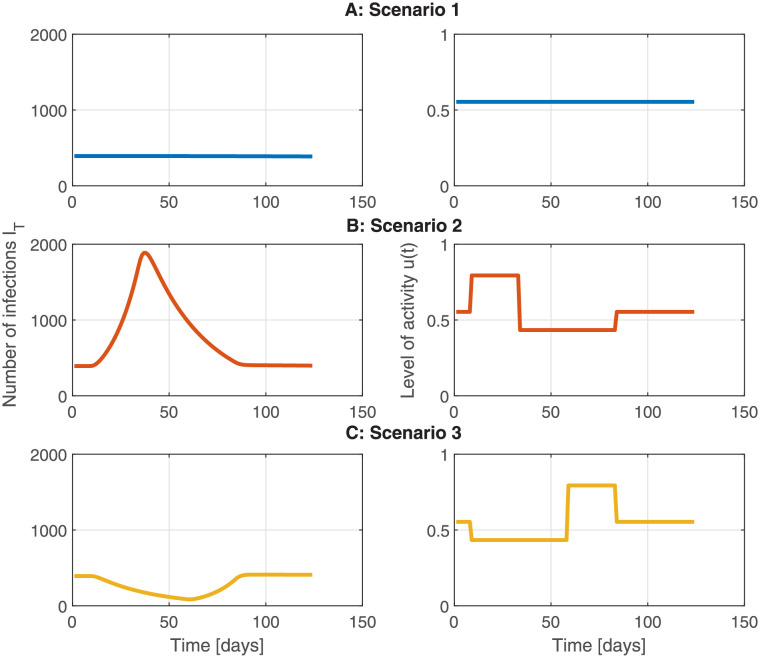
Pandemic sensitivity to interventions: Here we compare three approaches to pandemic control, holding all else equal (the cumulative interventions and the initial case rates, leading to the same final case rate). In the first scenario, interventions that lead to zero growth from the initial *I*_*T*_ = 400 are maintained, leading to a cumulative infection case count of ∫*I*_*T*_(*t*)*dt* of 48516. In the second scenario, restrictions are initially relaxed, leading to pandemic growth. Tighter restrictions are then implemented to return to the initial number of infections *I*_*T*_, leading to almost twice the number of total infections: ∫*I*_*T*_(*t*)*dt* = 95174, illustrating the cost of delayed action. The third scenario starts by implementing stricter interventions followed by relaxation, resulting in ∫*I*_*T*_(*t*)*dt* = 34821, a reduction of 28% from the first scenario despite the same average level of restrictions.

Analysis of system properties is simplified by the input-output representation and the simplified COVID-19 model described in Section 2. It follows that the number of infections at a specific time *t*_*c*_ is a function of the initial case count and the cumulative interventions up to time *t*_*c*_ (see S3 Appendix). Fig 4 illustrates the outcome for three different sequences of interventions in the SEEIQR model (preset, not using feedback control): Starting from the same initial case count and holding the average transformed interventions *v*(*t*) at zero (i.e. the same cumulative interventions and total cost in terms of *v*(*t*)), the case counts at *t*_*c*_ = 125 days is the same. However, the total number of infections differs by over 100% depending on the timing of restrictions. Fewest cases are obtained by early interventions, a result that is in line with studies investigation optimal lockdown strategies [12]. This open-loop example illustrates the sensitivity of the pandemic to the timing of interventions, and it will be shown that closed-loop feedback control provides a more systematic framework for delivering the desired response.

### 3.2 Model uncertainty

**Fig 5:**
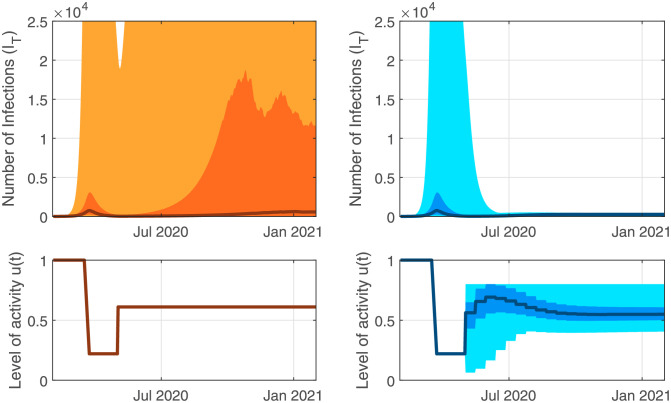
Accurate predictions are not required to design an effective policy to control the pandemic. Monte Carlo simulation results: The median is indicated with a thick line, the 25–75th percentiles and the min-max are indicated with shaded regions. Left: Under an open-loop policy, 40.25% of the 400 realizations reach a maximal number of infections over 13500 after June 30th, 2 months after the level of activity *u*(*t*) is increased. Right: With the same dynamic feedback policy in place starting May first for all realizations, and bi-weekly intervention updates, 100% of realizations maintain infections below 550 after June 30th. The variability in model parameters leads to adjustments in the interventions, which compensate for the differences between the models.

The effect of feedback on a highly uncertain pandemic is illustrated with Monte-Carlo simulations. Uncertainty in the parameter values is introduced by drawing parameters in the SEEIQR model from a range of values (see S1 Appendix) and simulating the dynamics, repeating this process for 400 realizations. Feedback control was based on hospital measured infections (Eq (2)) with an uncertain delay from infection to hospitalization *d* (mean of 14 days, range: 4—28 days), and *R*_0_ varied between 1.04 and 5.18 (see S1 Appendix for variability introduced in other parameters). The recommended controller used the tuning parameters *K*_*p*_ = 0.081, *K*_*i*_ = 4.4 ⋅ 10^−4^ in (6) (see S2 Appendix) and targeted a hospital-measured infected case count of 20 patients (approximately corresponding to a number of infections of 274). Fig 5 illustrates that including feedback (6) has effectively reduced the large uncertainty in projected case counts and transferred it to different levels of interventions. Here, we assumed that policy changes could be made only once every two weeks, discretizing the controller (6). We find that the feedback mechanism controls the pandemic despite the model uncertainty and broad range of time delays explored, in contrast to results presented by Casella [13]. This robustness is due to the near-linear form of the dynamics of the pandemic when measured on a logarithmic scale.

### 3.3 The impact of delays in measurement

**Fig 6:**
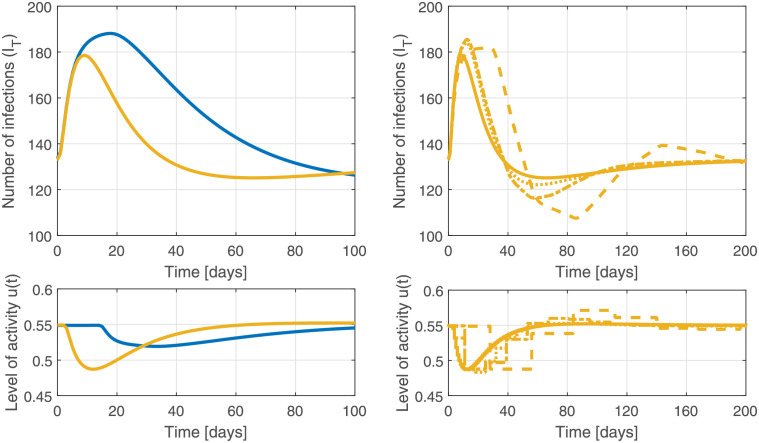
Minimizing delays in feedback improves performance: Left: Delays increase the time to detection of outbreaks and limit how aggressive policy responses can be. The top figure shows the response to an outbreak on day zero (100 exposed individuals). Under feedback with 14 days of delay (blue), the case load as a result of the outbreak is ∫*I*_*T*_(*t*)*dt* = 2022, compared to ∫*I*_*T*_(*t*)*dt* = 406 for feedback with two days of delay (yellow). The bottom figure shows the corresponding interventions. Reducing the delay also reduces the cumulative interventions required to control the outbreak by 6.7%. Right: The update frequency for interventions needs to be appropriate. When interventions are implemented with daily updates (yellow, as in the left panels), weekly updates (dotted) or updates every two weeks (dash-dotted), we observe similar levels of pandemic control. Updates to interventions every four weeks (dashed) introduce too much delay and cause oscillations.

We next compare scenarios based on measures with longer and shorter delays. In Scenario 1 (Eq (2)), feedback is based on counts from hospital testing, with a 14-day delay (controller parameters in (6): *K*_*p*_ = 0.081, *K*_*i*_ = 4.4 ⋅ 10^−4^. In Scenario 2 (Eq (3)), feedback is based on counts from rapid public testing, with a 2-day delay (controller parameters in (6): *K*_*p*_ = 0.20, *K*_*i*_ = 2.6 ⋅ 10^−3^). The outbreak is initialized with 100 exposed individuals on day zero. As illustrated in Fig 6, reducing the delays in measuring the disease burden from 14 days down to two days has the overall effect of reducing both the cumulative case count by 80% and the average level of interventions needed by 6.7%. Time delay is well-known to place fundamental (i.e., not addressable via controller tuning) limitations on the performance of a closed-loop system. Thus, to the extent possible, reducing or eliminating time delays will improve the performance of any pandemic response.

Discretization of these policy updates (i.e. using weekly, bi-weekly or monthly policy updates instead of daily updates) can be implemented with little loss in effective control, as long as the delay in policy updates is not too long with respect to the design objective (see Fig 6).

### 3.4 Varying public compliance and discrete interventions

**Fig 7:**
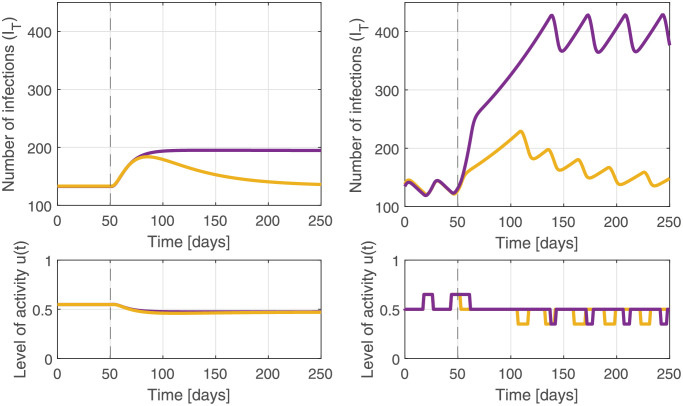
Feedback mitigates the effect of waning compliance to health directives. Left: After 50 days, the effectiveness of interventions decreases. Feedback control increases the level of interventions accordingly to stop pandemic growth. To fully counteract the (input) uncertainty, the cumulative error needs to be taken into account in the policy update (yellow; i.e., through the integrator term *K*_*i*_). If not (purple), the pandemic is stabilized at a sustained higher level of infections. Right: This effect is amplified if only a limited number of intervention levels are available. The area under the curve not using the cumulative error (purple) is 85% higher than when the cumulative error is used (yellow).

The effectiveness of non-pharmaceutical interventions has been hard to predict and may vary over time. Indeed, a key benefit of using feedback control is that appropriate action can be designed without knowing the exact effectiveness of these interventions. We simulated the case where the effectiveness of interventions was reduced by 0.075 on day 50 (Fig 7). Even though we did not adjust the control system in any way, pandemic control with feedback adjusts the interventions to return the case count to the target (orange curves). Importantly, this is achieved whether we allow for a continuous range of potential interventions (left) or a finite set (right). This robustness to changing public behaviour requires the use of an integral controller (*K*_*i*_), which takes the cumulative error into account. If, however, policy decisions were based only on current counts (no integral control: *K*_*i*_ = 0, holding proportional control *K*_*p*_ constant at 0.20), cases do not return to the target (purple) and are more sensitive to the responsiveness of policy interventions (right).

### 3.5 New variants and vaccinations

**Fig 8:**
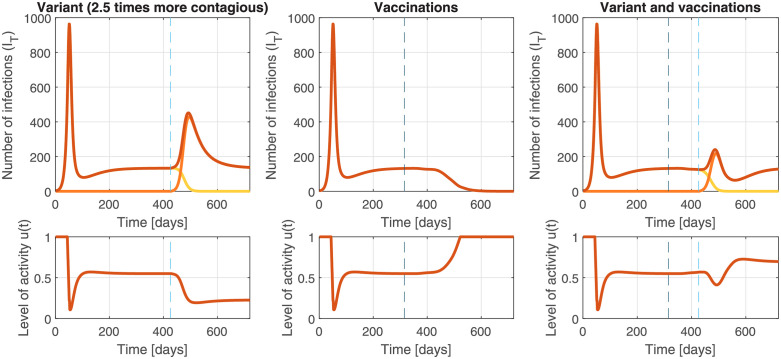
Feedback reduces the impact of the uncertainties inherent in a pandemic, such as the impact of vaccinations and new variants. Left: A new variant that is 2.5 times more infectious (*R*_0*v*_ = 7.5, introduced after 425 days indicated by a light blue dashed line) causes an initial increase in infections (dark orange line). Feedback control compensates with stricter measures that lead to extinction of the original strain (light line), while controlling the new strain at the target (orange line). Center: Feedback effectively reduces restrictions in response to observed declines in cases following a vaccine roll-out (start indicated by a dark blue dashed line), even when vaccine efficacy is unknown. Right: Feedback effectively responds to the combined effect of new variants and vaccine roll-out. In this simulation, vaccination of 70.9% of the population with a vaccine that is 95% effective is insufficient to stop the more transmissible variant on its own. The feedback policy adjusts restrictions, and the number of infections remains controlled at the target.

The effect of new, more contagious, variants and vaccinations is illustrated in Fig 8, again using a controller that tracks cases (Scenario 2 with a two-day delay). A variant that is 2.5 times more contagious is introduced 425 days into the pandemic, reflecting the arrival of the Delta variant in March 2021 in British Columbia, with an estimated *R*_0_ = 7.5 [14, 15] (note that this is beyond the transmissibility considered in Section 3.2). Vaccination, with 95% efficacy against transmission, is introduced according to the vaccination rates in British Columbia, starting in the week of December 19 2020 [16]. Vaccinations up to July 10th 2021 included in the simulations but without changing the controller (i.e., the interventions were blinded to the reason for the decreasing cases), with a final vaccination rate of 70.92% of the population. The susceptible population was reduced by *p*_*vac*_ equal to the rate of first dose vaccinations in BC, delayed by 21 days to account for the maturation of the immune system. Importantly, the performance of the controller in Fig 8 was achieved without any information about variants or vaccines, using measured case counts only. If such variant information is available earlier, the peak may be further reduced by augmenting the feedback using that information in a feedforward fashion, i.e., by taking action earlier, such that interventions are tightened or loosened proactively in anticipation of the arrival of a variant or vaccine, respectively.

## 4 Discussion

Decision making in public health to control COVID-19 relies heavily on modeling and prediction, putting both the influence and limitations of epidemiological modeling into the public eye. Decisions were made based on highly uncertain predictions, resulting in policies for which limited analysis tools are available (Fig 1a). Worst-case scenarios were often avoided by responding to observed hospitalizations or case numbers, bending down the epidemic curve through (implicit) feedback. Such reduction of the impact of uncertainty is one of the fundamental properties of feedback. In this work, we advocate for using feedback explicitly in the public health decision-making process (Fig 1b). A feedback-based approach can be implemented in public health by representing the decision-making problem explicitly in a feedback framework (Fig 1) and by expanding the currently used epidemiological modeling and prediction capacity with tools from feedback control theory. This shifts the focus from predictions to design of interventions and offers several advantages. By designing interventions using feedback, reliable decisions can be made in the face of uncertainty and even as the disease and social responses evolve, and there is less reliance on the accuracy of models because adjustments are made as trends diverge from public health objectives.

We show that a well-designed feedback policy can help mitigate the impacts of unknown and unknowable sources of uncertainty during a pandemic, including uncertainty in the dynamics, changing public compliance to recommended interventions, uncertainty about parameter values for a new disease, uncertainty about which interventions will work and how well, and the appearance of new variants or vaccines. All of these impacts are at best only roughly understood when they occur. While accurate models are more helpful than inaccurate models, these simulations illustrate that very simple feedback-based policies designed using an inaccurate and simple linear model approximation can successfully control a much more sophisticated SEEIQR model of the COVID-19 pandemic over a wide range of realistic conditions. Simulations also show that those feedback policies essentially transfer the uncertainty and variability in the case load to adjustments in the interventions. In nontechnical terms, if the case load starts to increase (decrease) for any reason, then the policy tightens (loosens) interventions in order to maintain the case load at its target (setpoint). The uncertainty-mitigating properties of feedback are fundamental in control theory.

Throughout the world, disease impacts through case counts or hospital load have been used to adjust restrictions. As discussed here, such feedback loops, using current values only (*K*_*i*_ = 0), are less efficient at controlling a disease than integral methods that also account for recent trends (Eq (6)). Integration allows information to accumulate that disease impacts are moving beyond a target, allowing earlier action and improved control (Fig 7).

With feedback control, any significant trend away from a target leads to a shift in policy, reducing variability in the quantity to be controlled (e.g., the case load). As a consequence, an effective policy can be designed even when the dynamics are uncertain; there is no need to wait for the development of a high-fidelity model. Of course, such models are useful to validate the performance of the feedback policy in simulations. As an added benefit, any changes that cause a shift in the controlled variable (e.g., new variants, changing behaviour, vaccination) lead to automatic adjustments in the interventions. Such adjustments reduce the total burden of a disease, while also minimizing unnecessary restrictions.

Representation of pandemic control explicitly in a feedback framework emphasizes characteristics that make pandemic control difficult: What happens if there are delays? How do we design interventions to achieve a desired level of activity and how do we know the impact of specific interventions on the spread of the virus? How do changes in policy impact public confidence? Public health officials face these challenges whether they use feedback control tools or not. In a feedback control framework, it is evident that delays in the gathering of information or in responding to that information limit the performance of a pandemic controller (Fig 6). Indeed time delays represent a fundamental limitation in closed-loop control systems [17]. An illustration of this problem is the recent landing on Mars of the Perseverance rover. The communication delay between Earth and Mars (7 min at the time of the landing), led NASA to coin the phrase “7 minutes of terror” as the delay made it impossible to control the descent from Earth. Time delays have to be explicitly included when designing feedback, lest the system becomes oscillatory or even unstable. In addition, as uncertainty in delays generally exists, robustness to delay variation has to be built in as well. Minimizing delays in the chain of measurement, reporting, decision making, and communication of restrictions is thus of critical importance. With smaller delays it is possible to reduce both the cumulative case counts as well as the cumulative severity of interventions. Rapid antigen testing and wastewater testing [18] are examples of methods that can detect outbreaks early and so are important tools to consider in pandemic control.

Independent of the tools used for public health decision making, the impact of specific interventions is at best partially known as there will initially be little data about impacts on transmission. The explicit feedback framework emphasizes the need to translate the control signal obtained by the controller into concrete, feasible public health interventions. Input from epidemiologists, public health experts, and behavioural scientists will remain necessary to define and refine the array of measures that can be used to control a pandemic with increasing stringency, taking into account the social and economic cost of individual measures. While ideally there would be a large set of measures implemented immediately, we have shown that pandemic control remains strong even when there are a limited set of measures (Fig 7) and only periodic adjustments (Fig 6).

A multitude of modeling and prediction tools are currently available to inform public health decision making. Similarly, a wide range of feedback control tools exists. This work used one of the simplest control algorithms available, the PI controller as suggested in Stewart et al. [19]. Many other, more sophisticated control algorithms have been proposed since the COVID-19 pandemic started. One such class of control algorithms is the so-called model-based predictive control or MPC (see e.g. Köhler et al. [20]), which is one of the most studied control methods for this problem [21, 22]. The theoretical advantage of MPC is that it can handle constraints, e.g., hard limits on variables such as interventions or the number of hospital or ICU beds. However, in order to achieve that in practice, MPC requires accurate models and measurements. Another potential advantage of MPC is that, because it is a multivariable optimization-based control technique, the performance index to be minimized can be tailored to include not only public health indicators but socio-economic ones as well, thus allowing an adjustment of the compromise between managing the pandemic and preserving the economy. Nevertheless, for broader understanding and widespread application, the simple PI controller explored here brings many of the advantages of feedback control, without needing detailed and accurate knowledge of disease dynamics, which is often unknowable during a pandemic.

The control community was fast to realize that its tools could be used to help manage the pandemic [23, 24]. Despite that, there has not been widespread use of feedback control theory by the public health and epidemiology community to guide decisions about when and how to enact public policies to manage the pandemic. This may be due to the fact that most of that work was published in control journals, typically not read by the public health community. We have aimed through this piece to raise awareness about control techniques in the public health community and to forge collaborations between the two fields.

## Supporting information

S1 Appendix SEEIQR model for COVID-19 in British Columbia. (ZIP)

S2 Appendix Controller tuning: Simple internal model control (SIMC). (ZIP)

S3 Appendix Analytical results: SIR model. (ZIP)
