# Supplementary material for: Effective pandemic policy design through feedback does not need accurate predictions
Source: PLOS Glob Public Health. 2023 Feb 3;3(2):e0000955. doi: 10.1371/journal.pgph.0000955 (PMC10021468; doi:10.1371/journal.pgph.0000955)
Supplement: S1 Appendix — (ZIP) [file pgph.0000955.s001.zip › S1_Appendix.pdf]

S1 Appendix. SEEIQR model for COVID-19 in British Columbia

Simulation results are based on a two-group compartmental SEEIQR model, developed to estimate the effect of social distancing in early 2020 in British Columbia, Canada [5].

This model contains two groups, one representing individuals who are socially distancing and one representing individuals who cannot distance, for example due to their profession. Individuals can switch groups, moving to the distanced group at rate m_d and to the non-distanced (relaxed) group at rate m_r. While all individuals may contact one another, distancing reduces the contact rate for individuals, both when infected and susceptible. The model compartments represent the number of susceptible (S), exposed (E1), pre-symptomatic and infectious (E2), symptomatic and infectious (I), quarantined (Q), recovered or deceased (R) for each group. The recovered (R) group does not affect infection rates and is omitted in this work.

The effect of vaccinations is included by reducing the size of the susceptible group. Variants with a different reproduction number have been included by additional compartments for each group, introducing a new pandemic drawing from the same susceptible population. The S, E_1, E_2, I and Q compartments for the distancing group are indicated with subscript d, compartments for the variant are indicated with subscript v. This does not account for possible re-infections. The effect of non-pharmaceutical interventions is introduced in this model through $u(t)$, the level of activity, which can vary with time and affects the transmission from and to the distanced group. Public health policies are assumed to impact $u(t)$ with $u(t) = 0$ representing complete restrictions resulting in no transmission involving the distanced group and $u(t) = 1$ representing no restrictions. For the original COVID-19 strain (with (t) omitted for readability), the equations for the group who are not physically distancing are:

$$
\begin{aligned}
\frac{dS}{dt} &= -\beta[I + E_2 + u(I_d + E_{2d})]\frac{S}{N} - \beta_v[I_v + E_{2v} + u(I_{dv} + E_{2dv})]\frac{S}{N} \\
&\qquad - m_d S + m_r S_d - p_{vac} N \frac{S}{S + S_d} \\
\frac{dE_1}{dt} &= \beta[I + E_2 + u(I_d + E_{2d})]\frac{S}{N} - k_1 E_1 - m_d E_1 + m_r E_{1d} + e_{outbreak} \\
\frac{dE_2}{dt} &= k_1 E_1 - k_2 E_2 - m_d E_2 + m_r E_{2d} \\
\frac{dI}{dt} &= k_2 E_2 - qI - I/D - m_d I + m_r I_d \\
\frac{dQ}{dt} &= qI - Q/D - m_d Q + m_r Q_d.
\end{aligned}
\tag{7}
$$

The transmission parameter, β, is related to the reproductive number, R_0, according to $\beta = \frac{R_0}{D+1/k_2}$ and $\beta_v = \frac{R_{0v}}{D+1/k_2}$ for the original strain and the variant respectively. N is the size of the population, with movement between the distancing and non-distancing groups having equilibriated so that $m_d/(m_r + m_d)$ of the cases are initially in the distancing group.

p_{vac} is the fraction of the population vaccinated per day, where we assumed daily vaccinations per day corresponding to the BC vaccine roll-out (assuming limited supply and/or vaccination capacity), rather than a proportional rate. k_1 is the rate of movement from the E_1 to E_2 compartment, k_2 from the E_2 to I compartment and q from the I to Q compartment. D is the mean duration of the infectious period. Outbreaks are simulated as imported cases ($e_{outbreak}$) in the E_1 compartment.

Analogous equations for the group that is distancing are:

$$
\begin{aligned}
\frac{dS_d}{dt} &= -u\beta[I + E_2 + u(I_d + E_{2d})]\frac{S_d}{N} - u\beta_v[I_v + E_{2v} + u(I_{dv} + E_{2dv})]\frac{S_d}{N} \\
&\quad + m_d S - m_r S_d - p_{vac} N \frac{S_d}{S + S_d} \\
\frac{dE_{1d}}{dt} &= u\beta[I + E_2 + u(I_d + E_{2d})]\frac{S_d}{N} - k_1 E_{1d} + m_d E_1 - m_r E_{1d} \\
\frac{dE_{2d}}{dt} &= k_1 E_{1d} - k_2 E_{2d} + m_d E_2 - m_r E_{2d} \\
\frac{dI_d}{dt} &= k_2 E_{2d} - qI_d - I_d/D + m_d I - m_r I_d \\
\frac{dQ_d}{dt} &= qI_d - Q_d/D + m_d Q - m_r Q_d
\end{aligned} \tag{8}
$$

The equations for the new variant are driven by the introduction of one or more variant cases $e_{variant}(t)$ at a specified time t:

$$
\begin{aligned}
\frac{dE_{1v}}{dt} &= \beta_v[I_v + E_{2v} + u(I_{dv} + E_{2dv})]\frac{S}{N} - k_1 E_{1v} - m_d E_{1v} + m_r E_{1dv} + e_{variant} \\
\frac{dE_{2v}}{dt} &= k_1 E_{1v} - k_2 E_{2v} - m_d E_{2v} + m_r E_{2dv} \\
\frac{dI_v}{dt} &= k_2 E_{2v} - qI_v - I_v/D - m_d I_v + m_r I_{dv} \\
\frac{dQ_v}{dt} &= qI_v - Q_v/D - m_d Q_v + m_r Q_{dv} \\
\frac{dE_{1dv}}{dt} &= u\beta_v[I_v + E_{2v} + u(I_{dv} + E_{2dv})]\frac{S_d}{N} - k_1 E_{1dv} + m_d E_{1v} - m_r E_{1dv} \\
\frac{dE_{2dv}}{dt} &= k_1 E_{1dv} - k_2 E_{2dv} + m_d E_{2v} - m_r E_{2dv} \\
\frac{dI_{dv}}{dt} &= k_2 E_{2dv} - qI_{dv} - I_{dv}/D + m_d I_v - m_r I_{dv} \\
\frac{dQ_{dv}}{dt} &= qI_{dv} - Q_{dv}/D + m_d Q_v - m_r Q_{dv}
\end{aligned} \tag{9}
$$

The nominal parameters used in the simulations are given in Table 1, as well as the range of parameters for the Monte-Carlo simulation presented in Section 3.2.

	D	k_1	k_2	q	R_0	Delay	R_{0v}	m_d	m_r
Nominal	5	0.2	1	0.05	3	14	7.5	0.1	0.02
Standard deviation	1	0.04	0.2	0.01	0.6	4	NA	0	0
Minimum	1.97	0.10	0.41	0.024	1.04	4	NA	NA	NA
Maximum	8.10	0.32	1.57	0.0784	5.18	28	NA	NA	NA

TABLE 1. Nominal model parameters and the range of values used to conduct Monte Carlo simulations. At the beginning of each of the 400 realizations, parameters were drawn from a normal distribution with average equal to the nominal values and standard deviations as indicated. The resulting models were used to simulate the pandemic with feedback control.

While the model (7)-(9) contains four groups (unable or able to socially distance, non-variant or variant),we assume that any measure of infections or hospitalizations represents the total of these groups: $I_T(t) = I(t) + I_d(t) + I_v(t) + I_{dv}(t)$.
