# Supplementary material for: Effective pandemic policy design through feedback does not need accurate predictions
Source: PLOS Glob Public Health. 2023 Feb 3;3(2):e0000955. doi: 10.1371/journal.pgph.0000955 (PMC10021468; doi:10.1371/journal.pgph.0000955)
Supplement: S2 Appendix — (ZIP) [file pgph.0000955.s002.zip › S2_Appendix.pdf]

## S2 Appendix. Controller tuning: Simple internal model control (SIMC)

In this work, we use a simple proportional-integral (PI) feedback controller (6), tuned using SIMC (simple internal model control). PI and PID (proportional-integral-derivative) controllers are used in the majority of industrial control applications, and a multitude of tuning methods exist. The SIMC rules were developed for process control applications, and presented as "Probably the best simple PID tuning rules in the world" [7]. Process control applications often involve complex and nonlinear processes for which accurate models may not exist. The SIMC rules offer a practical solution for such problems, where a simple linear model is used to approximate a complex system and to identify appropriate parameters for feedback control. COVID-19 exhibits complex, nonlinear behaviour, yet can be approximated with a simple approximate model, and therefore the SIMC method can be used to tune a feedback controller.

The first step in SIMC is to find a linear first- or second-order plus time delay model that approximates the system dynamics. Section 2.3.2 describes the proposed model approximation, which is then given by:

$$\frac{d^2z(t)}{dt^2} = A\frac{dz(t)}{dt} + B \cdot v(t-d) \tag{10}$$

The parameter values, $A = -0.5787$ and $B = 0.1572$, are derived using a least squares approximation to the nonlinear SEEIQR model (7) with nominal parameters in Table 1. These parameters are thus tuned to this pandemic model for British Columbia. The parameters $A, B$ and $d$ determine the time it takes for the system to respond (the delay $d$), the time constant to reach the constant slope, $\tau_2 = -1/A$, and the slope of the response, also referred to as the gain of the system, $k = -B/A$.

The second step in SIMC involves defining the controller (the function that maps $z(t)$ to the intervention variable $v(t) = u(t) - u_0$), with controller parameters determined by the approximate linear dynamics. For a system where the time delay $d$ is larger than the time constant $\tau_2$, the SIMC rules [7] recommend a proportional-integral control policy (6), repeated here for readability.

$$v(t) = K_p \cdot e(t) + K_i \cdot \int_{t_0}^{t} e(\tau)d\tau \tag{11}$$

The controller parameters can then be calculated according to:

$$\begin{aligned} \theta &= d + \tau_2 = d - 1/A \\ K_p &= \frac{1}{k}\frac{1}{\tau_c + \theta} = \frac{-A}{B}\frac{1}{\tau_c + \theta} \\ K_i &= \frac{K_p}{4(\tau_c + \theta)} \end{aligned} \tag{12}$$

The SIMC tuning rules introduce a single tuning parameter $\tau_c$ that defines the aggressiveness of the controller, by which we mean that a small value of $\tau_c$ corresponds to an aggressive controller with a fast speed of response, while a large value of $\tau_c$ corresponds to a more conservative controller with a slower response, better stability and increased robustness to uncertainty in the model. In this work, we chose $\tau_c = 30$ days for feedback with a long delay ($d = 14$ in Scenario 1) and $\tau_c = 15$ for a 2-day delay ($d = 2$ in Scenario 2). The controller parameters are then calculated to be $K_p = 0.081$, $K_i = 4.4 \cdot 10^{-4}$ for feedback with 14-day delay (2), and $K_p = 0.20$, $K_i = 2.6 \cdot 10^{-3}$ for the feedback with 2-day delay (3).
