# Supplementary material for: Effective pandemic policy design through feedback does not need accurate predictions
Source: PLOS Glob Public Health. 2023 Feb 3;3(2):e0000955. doi: 10.1371/journal.pgph.0000955 (PMC10021468; doi:10.1371/journal.pgph.0000955)
Supplement: S3 Appendix — (ZIP) [file pgph.0000955.s003.zip › S3_Appendix.pdf]

S3 Appendix. Analytical results: SIR model

The linearization described in Section 2.3.2 is an approximation of the dynamics of the nonlinear SEEIQR model (Appendix 4). For the special case of a simpler epidemiological model with an SIR structure with only one group (i.e. all individuals are socially distancing), this linearization is exact.

The SIR model, including social distancing measures, can be described as:

$$\begin{aligned}\frac{dS}{dt} &= -u(t)\beta I(t)S(t)/N,\\ \frac{dI}{dt} &= u(t)\beta I(t)S(t)/N - \gamma I(t),\\ \frac{dR}{dt} &= \gamma I(t),\end{aligned} \tag{13}$$

where $S(t), I(t)$ and $R(t)$ are the susceptible, infected, and recovered states respectively, N the total population, β the transmission parameter, and γ the recovery rate. The effect of social distancing is introduced as a factor $u(t)$ that affects transmission, i.e. transmission at time t equals $\beta(t) = u(t)\beta$, with $u = 1$ corresponding to no distancing and $u = 0$ corresponding to reduction of transmission to 0. For simplicity of notation, we assume the measured variable $y_c(t) = p \cdot I(t)$.

Without social distancing, this nonlinear SIR model can be linearized by assuming a constant susceptible population, at least over the short term or when infections remain low ($S(t) = S_0$), or normalized constant susceptible population ($S_0/N = 1$). The normalized infected population is then given by:

$$\frac{dI}{dt} = u(t)\beta I(t) - \gamma I(t), \tag{14}$$

The input-output relation between interventions $u(t)$ and $y_c(t) = p \cdot I(t)$ remains nonlinear.

Assuming that a constant fraction p of the active infections is detected, the transformed variable $z(t) = \ln(pI(t))$ is:

$$\frac{dz(t)}{dt} = \frac{d\ln(pI(t))}{dt} = \frac{d\left(\ln(p) + \ln(I(t))\right)}{dt} = \frac{d\ln(I(t))}{dt}. \tag{15}$$

Equation 15 demonstrates why feedback is insensitive to the proportion, p, of detected cases. This result does not depend on the model structure (SIR or SEEIQR) and is essential for decision making and for updating $v(t)$ and $u(t)$ appropriately, given incomplete information. It does not matter what proportion of cases is captured in the feedback, and it is not even necessary to know what the captured proportion is, because only proportional changes in the measured variable matter.

The proposed log transformation of the feedback measure, $z_c(t) = \ln(y_c(t))$, and affine transformation of the activity level $v(t) = u(t) - u_0$ linearize the input-output model for the SIR model:

$$\frac{dz_c(t)}{dt} = \frac{d\ln(I(t))}{dt} = \frac{1}{I}\frac{dI(t)}{dt} = u(t)\beta - \gamma = v(t)\beta, \tag{16}$$

with $u_0 = \gamma/\beta$. This result is now insensitive to scaling.

For the simple SIR model, the linearization is exact and the linearized model corresponds to a first order model. In a more complex epidemiological model such as the SEEIQR model, the linearization is approximate due to the exposed compartment, which also introduces additional dynamics that can be linearized by a lag as described in Section 2.3.2[iii]. The SEEIQR model considered in this study contains multiple groups, which introduces a second source of non-linearity through the factor $u(t)^2$. The simulation experiment included a series of step changes to the intervention $u(t)$ to ensure it covers a representative range of responses to which we fit the linear model.

When solving the differential equation described by (16), it follows that the number of infections at any time t_c is a function of the initial case count and the cumulative (integral of) interventions up to time t_c. Let L_0 represent $\ln(I_0)$, with I_0 the number of infected individuals at time t_0. The number of infected individuals at time t_c is then given by:

$$\ln(I(t_c)) = L_0 + \int_{t_0}^{t_c} v(t)\beta\, dt. \tag{17}$$

[iii]Equation (4) is equivalent to $A_2\frac{d^2z(t)}{dt^2} = A_1\frac{dz(t)}{dt} + B \cdot v(t-d)$ with $A_2 = 1$ and $A_1 = A$, while (16) corresponds to $A_2 = 0$, $A_1 = -1$, and $d = 0$.
